# Reduction in Urinary Arsenic with Bottled-water Intervention

**Published:** 2006-09

**Authors:** Arun B. Josyula, Hannah McClellen, Tracy A. Hysong, Margaret Kurzius-Spencer, Gerald S. Poplin, Stefan Stürup, Jefferey L. Burgess

**Affiliations:** ^1^ Environmental and Occupational Health, Mel and Enid Zuckerman College of Public Health, University of Arizona, Tucson, AZ 85719, USA; ^2^ Dartmouth Trace Element Analysis Core Facility, Dartmouth College, Hanover, NH, USA

**Keywords:** Arsenic, Drinking-water, Bottled water, United States

## Abstract

The study was conducted to measure the effectiveness of providing bottled water in reducing arsenic exposure. Urine, tap-water and toenail samples were collected from non-smoking adults residing in Ajo (n=40) and Tucson (n=33), Arizona, USA. The Ajo subjects were provided bottled water for 12 months prior to re-sampling. The mean total arsenic (μg/L) in tap-water was 20.3±3.7 in Ajo and 4.0±2.3 in Tucson. Baseline urinary total inorganic arsenic (μg/L) was significantly higher among the Ajo subjects (n=40, 29.1±20.4) than among the Tucson subjects (n=32, 11.0±12.0, p<0.001), as was creatinine-adjusted urinary total inorganic arsenic (μg/g) (35.5±25.2 vs 13.2±9.3, p<0.001). Baseline concentrations of arsenic (μg/g) in toenails were also higher among the Ajo subjects (0.51±0.72) than among the Tucson subjects (0.17±0.21) (p<0.001). After the intervention, the mean urinary total inorganic arsenic in Ajo (n=36) dropped by 21%, from 29.4±21.1 to 23.2±23.2 (p=0.026). The creatinine-adjusted urinary total inorganic arsenic and toenail arsenic levels did not differ significantly with the intervention. Provision of arsenic-free bottled water resulted in a modest reduction in urinary total inorganic arsenic.

## INTRODUCTION

Exposure of humans to inorganic arsenic is associated with an increased risk of lung, bladder, skin and other cancers (1, 2). Although inorganic arsenic is found throughout the environment, drinking-water constitutes the most significant source of exposure for most populations. Moschandreas *et al*. have estimated the contribution of drinking-water to the overall daily arsenic exposure to be 35%. Exposure of inorganic arsenic through ingestion (food and water) also depends on certain demographic characteristics, such as age, race/ethnicity, and poverty level (3).

On 22 January 2001, the U.S. Environmental Protection Agency lowered the maximum contaminant level (MCL) allowable for arsenic in drinking-water in the public water systems, from 50 μg/L to 10 μg/L, beginning 23 January 2006 (4). Although this will reduce arsenic exposure of drinking-water from the public water systems that currently do not meet these standards, it will require significant expenditure for modifications to these systems. In addition, exposure at the new standard of the U.S. Environmental Protection Agency may still confer health risks. The costs involved in reduction of arsenic may not be a reasonable option for small municipal water systems or households using personal well-water. Hence, arsenic-free bottled water may be a safe alternative in these settings.

In this study, we compared baseline urinary and toenail arsenic in Ajo and Tucson, Arizona, USA and evaluated the impact of providing arsenic-free bottled water, for one year, on biomarkers of arsenic exposure in Ajo. The primary hypothesis of the study was that provision of bottled-water supplies would reduce arsenic exposure as measured by total inorganic arsenic species in urine and concentrations of arsenic in toenails.

## MATERIALS AND METHODS

### Study area and selection of households

Census blocks within census tracts were initially selected at random. Based on a probability proportional to size (PPS) sampling protocol (5), our goal was to recruit a maximum of five households per census block. Our inclusion criteria required at least three years of continuous residence in Ajo at the time of recruitment, age over 18 years, exclusive use of tap-water for drinking and food preparation, and no current smoking. Given our restrictive inclusion criteria, we had difficulty in recruiting the sufficient number of subjects using the PPS protocol in Ajo. Subsequently, we resorted to a census of the entire community. In Tucson, the five census tracts that most closely resembled the Ajo population in age distribution (median age=52 years) and percentage of Hispanic residents (38%) in the 2000 census were selected. Two of the five census tracts were randomly selected, and census blocks and households in these blocks were randomized. Again, we recruited up to five households per block. The majority of households in both the communities that did not meet the inclusion criteria reported extensive use of bottled water. Recruitment of households took place during June-August 2002 in Ajo and during July 2002—August 2003 in Tucson. Up to two eligible subjects from each household were recruited.

### Sample collection

At the time of home-visit, participants completed a questionnaire adapted from the National Human Exposure Assessment Survey regarding residential and occupational history, health status, smoking status, and source of water. Water samples were collected using two sterile 50-cc polypropylene conical vials. Cold water from kitchen faucet of participants was allowed to run for one minute before collection began. The water samples were stored at 4 ºC until analysis of arsenic.

First-morning void urine samples were collected on the day of appointment for sample collection from each subject. Two sterile 120-cc screw-top containers were provided for the collection of urine. These containers had previously been tested to ensure that they did not impart any measurable arsenic to stored liquid. Urine samples were processed within two hours of collection using the following protocol. The urine sample was re-peatedly inverted and swirled to re-suspend the cells into solution, and approximately 200 mL was equally aliquoted into four 50-mL vials and spun at 2,400 rpm for 12 minutes at room temperature. The supernatant was then transferred to two 15-mL conical vials, tempo-rarily stored at -20 °C, later transported to Tucson, and stored at -70 °C prior to analysis for arsenic. On the appointment day, subjects were provided toenail clippers and small paper envelopes and were instructed to cut their big toenails and other toenails of both feet after taking their morning bath and to keep the toenail samples in the envelope for collection by the field team.

### Analysis of arsenic: urine and water

An HPLC-ICP-MS speciation method was modified for the measurement of arsenic (6). The HPLC system consisted of an Agilent 1100 HPLC (Agilent Technologies, Inc., Palo Alto, CA) with a reverse-phase C18 column (Prodigy 3μ ODS-3, 150×4.60 mm; Phenome-nex, Torrance, CA). The mobile phase (pH 5.85) contained 4.7 mM tetrabutylammonium hydroxide, 2 mM malonic acid, and 4% (v/v) methanol at a flow rate of 1.2 mL per minute. Column temperature was maintained at 50 °C. An Agilent 7500a ICP MS with a Babington nebulizer was used as the detector. The operating parameters were as follows: *Rf* power—1,500 W; plasma gas flow—15 L per minute; carrier flow—1.2 L per minute; and arsenic was measured at *m*/*z* 75. For total arsenic, an ASX500 autosampler (CETAC Technologies, Omaha, NE) was used for introducing the samples into the Agilent 7500a ICP-MS. The operating parameters were as follows: *Rf* power—1,500 W; plasma gas flow—15 L per minute; and carrier flow—1.2 L per minute. The acqui-sition parameters were arsenic measured at *m*/*z* 75, terbium (IS) measured at *m*/*z* 159, points per peak were 3, dwell time for arsenic was 1.5 seconds, and the dwell time for terbium was 1.5 seconds. Differential analysis of unstable MMA III and DMA III metabolites was not possible. The sensitivity limits for arsenic species was pre-determined to range from 0.04 to 0.08 μg/L.

For this analysis, total inorganic arsenic was defined as the sum of As^3+^, As^5+^, and the methylated metabolites monomethylarsonic acid (MMA) and dimethylarsenic acid (DMA). Urine or water arsenic species with levels lower than the detectable limits were assigned values of one-half the limit of detection for that particular arsenic species. The creatinine levels were estimated in urine samples using the Jaffe reaction method in a microplate format (Quidel Inc., San Diego, CA). Urinary arsenic concentration was adjusted for creatinine by expressing its level as a ratio with creatinine (μg/g creatinine). In accordance with the recommendations of the World Health Organization (WHO), urine samples with inadequate (<30 mg/dL; n=9) or excessive (>300 mg/dL; n=1) concentrations of creatinine were not included in analyses (7).

### Analysis of arsenic: toenails

As part of a cooperative project involving the University of Arizona and Dartmouth College, toenails were sent to the Dartmouth College for analysis of arsenic. Each toenail sample was washed with acetone, Triton X-100, and water to remove external contaminants. Afterwards, the complete sample was weighed into a Teflon vial, one mL of concentrated HNO_3_ was added and capped, and the sample was digested under pressure at 30 ºC in a microwave oven. The resulting solution was added to 4 mL of water and stored at 5 ºC until analysis. For arsenic analysis, this solution was diluted an additional four times with water and was analyzed using an Agilent 7500c ICP-MS instrument with an Octapole reaction cell pressurized with helium gas to remove potential interference from ArCl on *m*/*z*=75, the mass of the arsenic ion. The detection limit was 0.005 μg/g.

### Intervention: water delivery

Bottled water from Sparkletts® was tested to determine levels of arsenic using methods similar to those described above. A water-cooler with a regular supply of bottled water was provided to each participating household from Ajo for one year. The participants were instructed to use it for drinking, cooking, and preparation of all foods for the entire year. At the end of the year, the study team would return to collect another urine sample. Water was delivered twice a month or according to the needs of participants. The participants were telephoned over the course of the year to ensure that they were using bottled water for cooking and drinking and that enough was being delivered for their needs. Samples of urine and bottled water were collected at the end of one year using the methods described above.

### Statistical analysis

Data were analyzed using SPSS 12.0 (Chicago, IL). Population characteristics were compared using *t*-tests and chi-square analysis. The Mann-Whitney U-test was used for evaluating the differences at baseline in total and speciated urinary arsenic measures between Ajo and Tucson. Pre- and post-intervention-speciated urinary arsenic and toenail arsenic levels among the Ajo participants were compared using Wilcoxon signed rank test for matched pairs. Spearman's rank correlation coefficient was used for evaluating correlations between levels of arsenic in water, urine, and toenails.

The Institutional Review Board at the University of Arizona approved the study.

## RESULTS

Forty subjects from 33 households in Ajo and 33 subjects from 30 households in Tucson, who met eligibility requirements, were enrolled in the study. Their demographic characteristics are presented in Table 1. There were no significant differences between the Ajo and the Tucson study subjects in terms of gender, age, age distribution, race/ethnicity, past smoking, pre-existing illness, or the highest level of education attained. The participants were predominantly female, aged over 60 years, with at least a high school education. Approximately, one-third of them from each town were Hispanic. During the intervention, four subjects in Ajo dropped out of the study.

**Table 1.** Characteristics of participants in Ajo and Tucson

Characteristics	Ajo (n=40)		Tucson (n=33)	
	No.	%	No.	%
Female	25	63	21	64
Age (mean±SD)	61.8±15.8		64.7±14.5	
Age-group (years)				
<60	16	40	13	39
60–80	18	45	15	46
>80	6	15	5	15
Race/ethnicity				
White/non-Hispanic	23	58	23	70
Hispanic	16	40	10	30
Other	1	2	0	0
Past smoker	18	45	12	40
Pre-existing illness				
Ulcer	2	5	2	6
Asthma	14	35	16	48
Diabetes	8	20	5	15
Education				
Less than high school	7	18	4	12
High school graduate	13	32	9	27
College and beyond	20	50	20	61

### Baseline arsenic in water and urine

At baseline, no subjects used bottled water either for cooking or for drinking. Table 2 shows that the mean total arsenic in tap-water in Ajo was 20.3 μg/L (range 10.8–27.6 μg/L) and was significantly higher than that in Tucson (mean 4.02 μg/L, range 0.8–9.5 μg/L, p<0.001). Arsenate (As^5+^) was the major contributor to total inorganic arsenic in water in both the towns, comprising approximately 75% and 95% of the total in Tucson and Ajo respectively. The baseline concentrations of total urinary arsenic and total urinary inorganic arsenic were significantly higher in Ajo than in Tucson (p<0.001 for both). When the analysis was restricted to individuals who did not consume arsenic-containing food items, including seafood or mushrooms, urinary arsenic concentration was still significantly lower in Tucson than in Ajo. For urinary As^3+^, As^5+^, and MMA, 10%, 36%, and 8% of the samples respectively were below the limit of detection. The ratio of either MMA or DMA over the sum of As^3+^ and As^5+^ and the ratio of DMA/MMA did not differ significantly between the towns. Forty-three baseline urine samples were determined to be valid for adjustment of creatinine, based on the recommendations of WHO for concentrations of creatinine (Ajo, n=14; Tucson, n=29). Twenty samples from Ajo were not available for analysis of creatinine. The population characteristics of the subset of individuals with valid measurements of creatinine were compared with the rest in each group, but no significant differences were observed. Baseline creatinine-adjusted total urinary inorganic arsenic was significantly higher in Ajo than in Tucson (p<0.001).

**Table 2.** Characteristics of arsenic species in subjects from Tucson and Ajo at baseline

Arsenic species or ratio	Ajo Mean±SD	Tucson Mean±SD	p value
Tap-water (n)	33	29	
Total arsenic (μg/L)	20.3±3.7	4.0±2.3	<0.001
Urine (n)	40	32	
Total arsenic (μg/L)	28.3±18.6	14.0±13.5	<0.001
Total inorganic arsenic (μg/L)	29.1±20.4	11.0±12.0	<0.001
Creatinine (μg/dL)*	108.2±57.4	87.5±50.8	0.254
Total inorganic arsenic/creatinine (μg/g)*	30.6±13.2	13.7±9.6	<0.001
As^3+^ (μg/L)	6.8±8.8	4.3±8.6	0.011
As^5+^ (μg/L)	2.0±2.6	0.2±0.5	< 0.001
MMA (μg/L)	2.9±3.7	1.0±1.1	< 0.001
DMA (μg/L)	17.5±12.5	5.5±4.4	< 0.001
MMA/(As^3+^+As^5+^)	0.7±0.6	1.9±3.2	0.461
DMA/(As^3+^+As^5+^)	5.1±5.9	10.8±14.1	0.225
DMA/MMA	13.1±29.0	14.4±23.1	0.511
Toenail (n)	38	33	
Arsenic (μg/g)	0.51±0.72	0.17±0.21	0.001

*Valid values n=14 for Ajo and n=29 for Tucson;

SD=Standard deviation

### Water intervention in Ajo and changes in speciated urinary arsenic

Concentrations of arsenic in bottled water were all below the limit of detection (∼0.1 μg/L). Comparison of total inorganic urinary arsenic concentrations at baseline and follow-up revealed a 21% reduction associated with the intervention (from 29.4 to 23.2 μg/L, p<0.026) (Table 3). There was no significant difference in creatinine-adjusted urinary total inorganic arsenic between the pre- and the post-intervention (n=11). Drinking-water intervention was associated with a significant reduction in DMA and DMA/MMA ratio. No significant changes in the relative levels of MMA/(As^3+^+As^5+^) or DMA/(As^3+^+As^5+^) were observed with the bottled-water intervention.

**Table 3.** Arsenic species in Ajo before and after bottled-water intervention

Arsenic species or ratio	Pre-intervention	Post-intervention	p value
Urine (n)	36	36	
Total arsenic (μg/L)	28.3±18.9	22.4±21.4	0.035
Total inorganic arsenic (μg/L)	29.4±21.1	23.2±23.2	0.026
Creatinine (μg/dL)*	109.2±58.3	107.9±86.2	0.424
Total inorganic arsenic/creatinine (μg/g)*	27.8±10.1	31.8±23.7	0.79
As^3+^ (μg/L)	6.4±9.0	10.3±20.8	0.730
As^5+^ (μg/L)	2.1±2.7	1.7±1.9	0.943
MMA (μg/L)	3.0±3.8	2.0±1.8	0.091
DMA (μg/L)	17.9±12.7	9.1±8.1	<0.001
MMA/(As^3+^+As^5+^)	0.7±0.6	1.2±3.5	0.814
DMA/(As^3+^+As^5+^)	5.3±6.1	3.8±5.2	0.114
DMA/MMA	8.3±5.2	5.0±3.3	0.001
Toenail (n)	34	34	
Arsenic (μg/g)	0.49±0.68	0.26±0.19	0.061

*Number of samples with available valid creatinine levels=11

As=Arsenic;

DMA=Dimethylarsenic acid;

MMA=Monomethylarsonic acid

When these comparisons were restricted to those 29 subjects who reported using bottled water for cooking, drinking, and beverage preparation, the total inorganic arsenic levels were significantly reduced with the intervention (31.0±22.9 vs 21.6±20.6 μg/L, p=0.013). How-ever, creatinine-adjusted urinary inorganic arsenic was not significantly reduced with the intervention (27.4±8.0 v. 27.3±24.4 μg/g, p=0.735). The relative proportions of MMA/(As^3+^+As^5+^) or DMA/(As^3+^+As^5+^) did not vary significantly with the intervention.

### Changes in toenail arsenic

Toenails were collected, on average, within one week of initial interview. At baseline, the toenail arsenic levels were significantly higher in Ajo than in Tucson (Table 2). When data from both the towns were combined, there was a significant positive correlation between toenail arsenic and total urinary inorganic arsenic (ρ_s_=0.344, p=0.004). There was a non-significant decline of approximately 47% between the pre- and the post-intervention toenail arsenic level (p<0.061) (Table 3). For the 28 subjects who provided toenail samples and reported exclusive use of bottled water for drinking and food preparation during the intervention period, a significant reduction in concentrations of arsenic in toenails was observed (0.53±0.75 μg/g vs 0.26±0.21 μg/g, p=0.032).

## DISCUSSION

As expected, we found significantly increased total arsenic and total inorganic urinary arsenic, along with significantly increased concentrations of arsenic in toenail in Ajo compared to Tucson. This finding was consistent with a higher exposure to arsenic in Ajo through tap-water. Arsenic-free bottled-water intervention in Ajo significantly reduced total inorganic arsenic in urine, although the change was not observed with adjustment of creatinine, and the decline in toenail arsenic was only significant when the analysis was restricted to those subjects who reported exclusive use of bottled water. These findings suggest that, over a one-year period, provision of bottled water had limited effectiveness in reducing arsenic exposure in this study community.

Although the total urinary arsenic levels in Ajo were reduced significantly with the bottled-water intervention, the post-intervention urinary arsenic levels were still far above those found in Tucson. This finding denotes that some degree of arsenic exposure continued to occur among the Ajo subjects despite the drinking-water intervention. Although 29 of the 36 subjects reported exclusive use of bottled water for cooking and drinking, the intermittent use of tap-water for cooking and preparation of beverages by the study subjects, and exposure to arsenic through food or other sources, may help explain the limited effect observed with the intervention.

Food and water are the major primary sources of non-occupational daily arsenic exposure. According to the toxicological profile of the U.S. Department of Health and Human Services for arsenic, food contributes up to 2.4 μg/kg body-weight of arsenic exposure (∼37% of which is inorganic), depending on the source and quantity. Similarly, arsenic exposure from water could range from as low as 0.1 to as high as 31 μg/kg of body-weight, depending on locality of the water source. Quality of soil and environmental air contributes a lesser extent to daily arsenic exposure (8). Although we could not quantify all these factors in our analysis, drinking-water intervention alone may not sufficiently reduce daily arsenic exposure, especially when the baseline daily exposure contribution from water is not high.

Very little published information is available on the effectiveness of provision of bottled water in reducing arsenic exposure. Hopenhayn-Rich *et al*. studied the effects of drinking-water intervention in individuals with high arsenic exposure (≥600 μg/L) (9). In the Hopenhayn-Rich study, replacement drinking-water, which contained arsenic at approximately 45 μg/L, was provided for two months. The mean urinary total inorganic arsenic level before and after drinking-water intervention was 636 μg/L and 166 μg/L respectively. Similar to our study, the authors found a reduction in both DMA but also found a reduction in MMA and inorganic arsenic. In contrast to our study, the DMA/MMA ratio increased following the intervention.

Baseline measures of both MMA and DMA were significantly higher in individuals in Ajo than in Tucson, but the ratio of either MMA/(As^3+^+As^5+^) or DMA/ (As^3+^+As^5+^) as respective indicators of primary and secondary arsenic methylation activity in the liver, did not differ significantly between the towns. However, the DMA/MMA ratio was significantly reduced with the intervention. Approximately, 70–85% of the ingested arsenic is excreted through the kidney with DMA being the principal metabolite. As reported in other studies, DMA is the principal arsenic metabolite following low level of arsenic exposure, trailed by MMA and inorganic forms (9, 10). Due to increased solubility and more rapid rate of excretion, methylated forms (MMA and DMA) are generally thought to be less acutely toxic than inorganic forms. However, in more recent reports, Aposhian *et al*. and Wildfang *et al*. suggested that arsenic methylation may not be the universal detoxification mechanism for arsenic as once thought (11, 12). Other studies have suggested that arsenic methylation may potentiate its toxicity (13, 14). Also, contrary to an earlier belief that inorganic arsenite is the most toxic form of arsenic, Petrick *et al*. described relative toxicity profiles in human liver cells in the following order: MMA (III) >>> As^3+^ >> As^5+^ >> MMA (V)=DMA (V) (15).

Our study had several limitations. Estimates of total arsenic exposure through diet, water, dust, and air were not available. Although increased exposure to environmental arsenic near copper-smelting towns has been well-documented (16), we were unable to assess the contribution of exposures at work or away from home. Another documented source of exposure to arsenic is through food, especially fish (3), and consumption of foods grown in arsenic-contaminated soils (17). However, measurement of inorganic arsenic concentration rather than the total arsenic concentrations should reduce confounding effects of seafood ingestion, and in a previous study in two Arizona mining towns, concentration of arsenic in house-dust was not associated with urinary arsenic concentrations in adults (18). Because urine creatinine levels were only available for 11 individuals from Ajo at baseline, our statistical power to detect pre- and post-intervention differences in creatinine-adjusted arsenic levels was limited.

In conclusion, the arsenic-free drinking-water intervention resulted in only modest reductions in urinary arsenic concentrations. The total inorganic urinary arsenic level was reduced by 21%, although analysis of crea-tinine-adjusted urinary and toenail arsenic failed to cor-roborate significant reduction in arsenic exposure. This implies that there was probably greater use of tap-water for food and beverage preparations than realized or reported and/or that other non-water sources of arsenic were present. Evaluations of similar populations before and after providing water-treatment systems, as contrasted with bottled water, would provide a useful comparison.

## ACKNOWLEDGEMENTS

The study was supported by NIEHS Superfund Basic Research Program grant no. P42 ES04940 and NIEHS Center for Toxicology: Southwest Environmental Health Sciences Center grant no. P30 ES06694.

Work should be attributed to: Environmental and Occupational Health, University of Arizona.
